# Periacetabular osteotomy provides durable correction and low arthroplasty conversion at  ≥ 7 years: prospective middle eastern study

**DOI:** 10.1007/s00264-026-06789-0

**Published:** 2026-04-09

**Authors:** Mahmoud Fahmy, Ahmed Hazem Abdelazeem, Mostafa Ahmed Shawky

**Affiliations:** Orthopaedic Department, Cairo University Kasr Alainy Faculty of Medicine, Cairo, Egypt

**Keywords:** Periacetabular osteotomy, Acetabular dysplasia, Hip preservation surgery, Long-term outcomes

## Abstract

**Background:**

Long-term prospective data on periacetabular osteotomy (PAO) from Middle Eastern populations are limited. This study evaluated ≥ seven year clinical, functional, and radiographic outcomes following PAO and identified predictors of survivorship.

**Methods:**

Thirty-six consecutive patients (34.6 ± 7.2 years; 78% female) undergoing PAO (2014–2018) were prospectively followed. Inclusion required symptomatic dysplasia with Tönnis 0–2. Outcomes included HHS, WOMAC, HOS, SF-36, radiographic parameters (LCEA, AI), complications, and THA conversion. Reliability, multivariate regression, and Kaplan–Meier analyses were performed.

**Results:**

At 7.8 ± 1.2 years, HHS improved from 63.5 ± 11.2 to 89.6 ± 7.8 (*p* < 0.001). LCEA increased from 16.2 ± 4.3° to 31.8 ± 3.9° and AI decreased from 22.8 ± 5.1° to 7.2 ± 3.6°. ICC for measurements was 0.92. Complications occurred in 16.7% (mostly minor). THA conversion was 5.6%, both with preoperative Tönnis 2 and correction < 12°. Magnitude of LCEA correction independently predicted HHS improvement (β = 0.41, *p* < 0.01).

**Conclusions:**

PAO achieved durable correction and sustained functional improvement with low THA conversion at mid- to long-term follow-up. Preoperative cartilage status and adequacy of correction are key determinants of outcome.

## Introduction

Developmental dysplasia of the hip (DDH) is a major cause of hip pain and functional limitation in young adults and represents a well-recognized precursor of early osteoarthritis. Insufficient acetabular coverage leads to abnormal joint mechanics and progressive cartilage overload, and up to 50–60% of symptomatic patients may require total hip arthroplasty (THA) by mid-adulthood if structural correction is not achieved**.** Nonoperative treatment may provide temporary symptom relief but does not address the underlying deformity, making joint-preserving surgery the cornerstone of management in appropriately selected patients [1–7].

Periacetabular osteotomy (PAO) is currently considered the standard joint-preserving procedure for symptomatic acetabular dysplasia with preserved or minimally degenerated cartilage. By reorienting the acetabulum while maintaining posterior column stability, PAO improves femoral head coverage and joint congruency, resulting in substantial pain relief and functional recovery. Most long-term evidence originates from North American and European cohorts, whereas data from Middle Eastern populations—who may differ in morphology, BMI distribution, and access to early referral—remain limited**.** Furthermore, predictors of durable correction and survivorship beyond mid-term follow-up are not fully defined in this region [8–15].

Long-term prospective data from Middle Eastern populations remain scarce, particularly regarding durability of correction and predictors of survivorship. This study aimed to evaluate ≥ seven year clinical and radiographic outcomes after PAO, determine factors associated with functional improvement and THA conversion, and assess measurement reliability. We hypothesized that PAO would provide sustained functional improvement with maintained radiographic correction and low THA conversion, and that preoperative joint status and magnitude of correction would be significant determinants of outcome.

## Patients and methods

This prospective cohort study was conducted on patients undergoing periacetabular osteotomy (PAO) for symptomatic acetabular dysplasia between 2014 and 2018. The study protocol was approved by the institutional ethics committee (N-269–2025), and all participants provided informed consent prior to inclusion. The research adhered to the principles outlined in the Declaration of Helsinki for medical research involving human subjects.

Patients were included if they presented with symptomatic acetabular dysplasia, preserved or minimally degenerated hip cartilage (defined as Tönnis grade 0–2), and a desire for joint-preserving surgery. In cases of bilateral dysplasia, only the first operated hip was included in the analysis to maintain independence of observations. Exclusion criteria comprised advanced osteoarthritis (Tönnis grade ≥ 3), previous hip arthroplasty, inflammatory arthropathies, neuromuscular disorders affecting hip biomechanics, or other conditions that could compromise surgical correction or follow-up reliability. Only patients with complete baseline and follow-up assessments of a minimum of seven years were analyzed.

All procedures were performed using the Bernese periacetabular osteotomy (Ganz PAO) technique, which preserves the integrity of the posterior column while allowing controlled reorientation of the acetabular fragment. This technique enables correction of acetabular coverage while maintaining pelvic stability and vascular supply to the acetabular fragment. The cases included in this study were performed by a specialized hip preservation team. These cases represent the surgeon’s clinical experience with Bernese PAO over a seven-year period rather than the initial learning phase, as the surgical team had established prior experience in hip preservation procedures before the initiation of the study cohort.

**Fig. 1:**
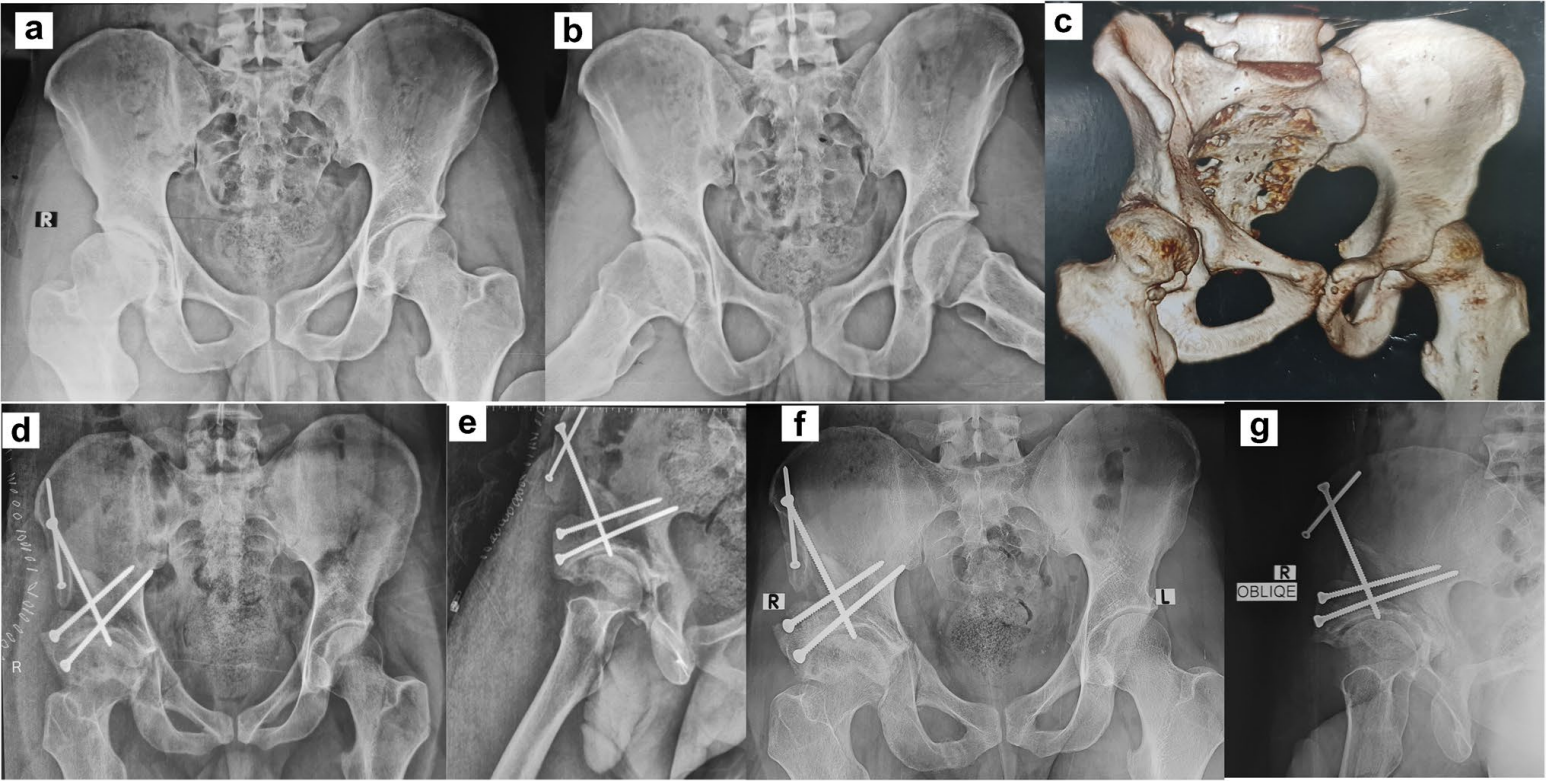
A 28-year-old male with symptomatic adult acetabular dysplasia, more pronounced on the right hip, treated with PAO. **a–c** Preoperative AP and lateral hip radiograph with 3D CT demonstrating reduced lateral center–edge angle (LCEA) and increased acetabular index (AI) consistent with acetabular dysplasia. **d**, **e** Immediate postoperative AP pelvic and lateral hip radiographs showing adequate acetabular reorientation with correction of both LCEA and AI, stabilized using four 4.5-mm cortical screws. **f**, **g** Final follow-up radiographs at 7 years demonstrating maintained acetabular correction, complete osteotomy union, stable fixation, and absence of secondary complications

**Fig. 2:**
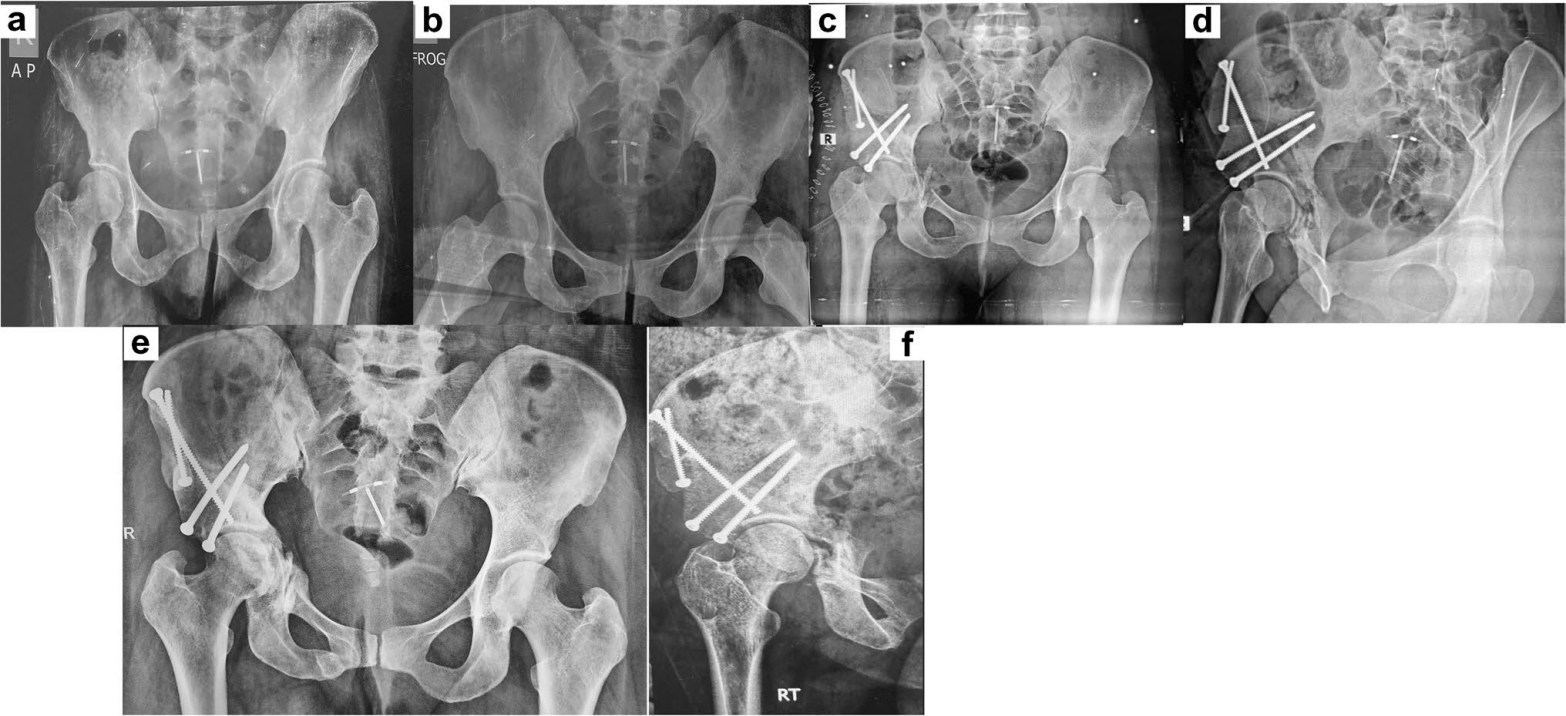
A 29-year-old female with symptomatic adult acetabular dysplasia, predominantly affecting the right hip, treated with PAO. **a**, **b** Preoperative radiographs including demonstrating acetabular dysplasia with reduced LCEA and increased AI. **c**, **d** Immediate postoperative radiographs showing adequate acetabular reorientation with correction of both LCEA and AI, stabilized using four 4.5-mm cortical screws. **e**, **f** Final follow-up radiographs at 8 years demonstrating maintained correction, complete osteotomy union, stable fixation, and absence of secondary complications

All osteotomies were performed through a single modified Smith–Petersen approach, which provided adequate exposure for the pubic, ischial, and supra-acetabular cuts required for the Bernese PAO. Sequential osteotomies were performed following the principles of the Bernese PAO. The ischial osteotomy was created through the infracotyloid groove, allowing safe mobilization of the acetabular fragment while preserving the posterior column. The pubic osteotomy was performed just medial to the iliopectineal eminence at the highest point of the superior pubic ramus. A supra-acetabular iliac osteotomy was then completed to allow controlled acetabular reorientation. This configuration permitted three-dimensional correction of acetabular coverage while maintaining pelvic stability and preserving the posterior column. Intraoperative fluoroscopy was used to guide correction and assess acetabular orientation, targeting restoration of physiological lateral center–edge angle and acetabular index. Fixation was achieved using three to five cortical screws per patient, and intraoperative fluoroscopy confirmed alignment and coverage. Criteria for additional acetabular correction included residual lateral center–edge angle (LCEA) < 25° or acetabular index (AI) > 15° intraoperatively. [Fig 1,2,and 3 are case examples] Soft tissue management included meticulous preservation of periarticular musculature and capsular integrity to facilitate early rehabilitation. Intraoperative complications, including temporary neuropraxia or minor fractures, were recorded.

Postoperative rehabilitation followed a standardized protocol. Patients were maintained on partial weight bearing with crutches for the first six to eight weeks, followed by gradual progression to full weight bearing after radiographic evidence of osteotomy healing. Passive and active-assisted range-of-motion exercises were initiated during the first postoperative week. Structured physiotherapy emphasizing hip abductor strengthening and gait training was introduced at approximately six to eight weeks, with gradual return to low-impact activities after three months and unrestricted daily activities typically permitted after four to six months. Clinical follow-up was scheduled at six weeks, three months, six months, one year, and annually thereafter for a minimum of seven years, including standardized assessments of pain, function, and quality of life. Rehabilitation adherence was documented as full, partial, or non-adherent to evaluate its impact on outcomes.

Radiographic evaluation included standardized anteroposterior pelvic and lateral hip radiographs obtained preoperatively, immediately postoperatively, and at each follow-up. Parameters assessed included lateral center–edge angle (LCEA), acetabular index (AI), and acetabular coverage angles to quantify correction and monitor maintenance over time. Radiographic measurements were performed using calibrated digital PACS measurement software. To minimize the influence of pelvic tilt, standardized anteroposterior pelvic radiographs were obtained with the coccyx positioned approximately 1–3 cm above the pubic symphysis, consistent with accepted radiographic criteria for neutral pelvic positioning. Radiographs were calibrated using standardized markers, and inter- and intraobserver reliability was assessed with intraclass correlation coefficients (ICC). Osteoarthritis progression was graded using the Tönnis classification, and target postoperative correction was defined as LCEA 30–40° and AI 0–10°.

Functional outcomes were assessed using validated patient-reported outcome measures (PROMs), including the Harris Hip Score (HHS), Western Ontario and McMaster Universities Osteoarthritis Index (WOMAC), Hip Outcome Score (HOS), and SF-36 physical component. Objective evaluations included hip range of motion, gait analysis, and standardized physical function tests. Minimal clinically important difference (MCID) thresholds were applied to contextualize the clinical relevance of observed improvements.

Statistical Analysis: Statistical analyses were performed using SPSS Statistics (Version 26, IBM Corp., Armonk, NY, USA). Continuous data are reported as mean ± SD and categorical data as counts (%). Longitudinal changes were assessed with repeated-measures ANOVA or linear mixed-effects models (Bonferroni-adjusted). Correlations between radiographic correction and functional outcomes used Pearson or Spearman coefficients. Multivariate regression identified predictors of outcomes and THA conversion (age, sex, BMI, Tönnis grade, correction magnitude). Significance was set at p < 0.05, with sufficient power to detect moderate effects.

## Results

The study included 36 patients with symptomatic acetabular dysplasia who underwent PAO. The mean age was 34.6 ± 7.2 years (range 19–49), with 78% female. Mean BMI was 24.3 ± 3.1 kg/m^2^ (range 19.8–31.5). Preoperative cartilage was preserved in 72% and minimally degenerated in 28% (Tönnis 0–2). Among patients < 40 years, cartilage grades were 0 (52%), 1 (36%), and 2 (12%), while those ≥ 40 years had 0 (18%), 1 (55%), and 2 (27%), *p* = 0.15; baseline demographics and joint status did not differ by age or cartilage subgroup. Mean operative time was 142 ± 25 min (range 110–190) and blood loss 420 ± 85 mL (range 300–600), with moderate correlation between the two (r = 0.48, *p* = 0.002). Additional acetabular correction was associated with longer surgery (155 ± 22 vs. 138 ± 20 min, p = 0.01, d = 0.78). Mean follow-up was 7.8 ± 1.2 years (range 7–10), with 92% completing all assessments; follow-up adherence was consistent across subgroups.

Radiographic evaluation showed substantial and largely maintained correction at final follow-up. LCEA improved from 16.2 ± 4.3° preoperatively to 32.5 ± 3.6° postoperatively, remaining 31.8 ± 3.9° at last follow-up (Δ =  + 15.6°, 95% CI 14.1–17.1°). Acetabular index decreased from 22.8 ± 5.1° to 6.5 ± 3.4° postoperatively and 7.2 ± 3.6° at last follow-up (Δ =  − 15.6°, 95% CI − 17.2 to − 14.0°). Acetabular coverage angles increased from 55.1 ± 6.2° to 72.3 ± 5.4° postoperatively and remained 71.5 ± 5.6° at final follow-up (Δ =  + 16.4°, 95% CI 14.8–18.0°). Interobserver reliability was excellent (ICC = 0.92, 95% CI 0.88–0.95), and lower preoperative Tönnis grades were associated with greater correction (*p* = 0.003).All functional scores improved markedly. HHS increased from 63.5 ± 11.2 to 89.6 ± 7.8 (Δ =  + 26.1, 95% CI 22.4–29.8), WOMAC decreased from 42.1 ± 9.5 to 14.3 ± 6.2 (Δ =  − 27.8, 95% CI − 31.2 to − 24.4), HOS improved from 61.4 ± 10.7 to 86.7 ± 8.1 (Δ =  + 25.3, 95% CI 21.9–28.7), and SF-36 physical score rose from 38.2 ± 7.4 to 52.7 ± 5.6 (Δ =  + 14.5, 95% CI 12.1–16.9). LCEA correction correlated with HHS improvement (r = 0.51, *p* < 0.001), and AI correction correlated with WOMAC improvement (r = 0.46, *p* = 0.002).

Subgroup analysis demonstrated that patients aged ≥ 40 years achieved comparable improvement from baseline (ΔHHS 25.3 ± 7.2, p = 0.54), although their absolute final HHS was slightly lower than younger patients (87.2 ± 6.9 vs. 90.3 ± 7.2, *p* = 0.24). Multivariate regression adjusting for preoperative cartilage status and Tönnis grade confirmed that age was not an independent predictor of ΔHHS (β =  − 1.8, *p* = 0.21) (Table 1).

Overall complications occurred in 16.7% of patients (*n* = 6), with minor complications in 8.3% (*n* = 3), including transient lateral femoral cutaneous neuropraxia resolving spontaneously (5.6%, *n* = 2) and superficial wound infection managed nonoperatively (2.7%, *n* = 1). Major complications (8.3%, *n* = 3) included two conversions to THA (5.6%) for progressive osteoarthritis and one case of symptomatic screw prominence requiring hardware removal. Both THA conversions occurred in patients aged ≥ 45 years with preoperative Tönnis grade 2 and LCEA correction < 12°. No intraoperative major complications, malalignment, nonunion, or screw-related issues were observed. Higher preoperative Tönnis grade correlated with overall complication rate (r = 0.42, *p* = 0.01).

## Discussion

In this prospective cohort of Middle Eastern patients with symptomatic acetabular dysplasia, periacetabular osteotomy (PAO) achieved durable functional improvement, precise radiographic correction, and a low mid-term THA conversion rate at a minimum follow-up of seven years. Patients experienced a mean HHS improvement with a large effect size (d = 2.3), accompanied by parallel gains in WOMAC, HOS, and SF-36 scores, confirming clinically meaningful benefits. The low complication burden and sustained outcomes in this regional cohort underscore the reproducibility of PAO results outside Western populations, despite subtle differences in BMI and hip morphology.

Our functional outcomes closely meet with established series. Swarup et al. reported significant HHS improvements in adolescent borderline dysplasia [1], while Kumar et al. and Chang et al. observed comparable PROM gains across minimally invasive and Bernese PAO approaches [2, 4]. These findings suggest that, when performed in carefully selected patients, PAO can achieve similar mid- to long-term functional recovery across diverse populations.

Radiographic correction was robust and durable, with significant improvements in lateral center–edge angle (LCEA) and acetabular index (AI) maintained throughout follow-up. Akbulut et al. previously demonstrated that sustained radiographic correction correlates strongly with long-term functional outcomes [11], a relationship reinforced by our cohort. The precision of acetabular reorientation, rather than absolute age or BMI alone, appears central to joint preservation, supporting the concept that mechanical restoration drives clinical durability.

The prognostic importance of preoperative cartilage status was evident. Both THA conversions occurred in hips with Tönnis grade 2 osteoarthritis and suboptimal correction, echoing prior observations by Tan et al. and Novais et al., who reported that patients without advanced degeneration maintain superior survivorship beyond ten years [6, 7]. Sensitivity analyses excluding these higher-risk hips confirmed that HHS and WOMAC improvements remained robust, reinforcing the predictive value of cartilage preservation in achieving durable outcomes.

Age remains a nuanced predictor. In our cohort, older patients achieved meaningful relative improvement, consistent with Novais et al. and Lee et al., who demonstrated that patients ≥ 40 years with preserved cartilage and good baseline function can still achieve effective hip preservation [7, 10]. Absolute scores may be slightly lower in older patients, but the magnitude of functional gain and pain relief remains clinically relevant, emphasizing that biological joint age outweighs chronological age in decision-making. An additional point of interest in this cohort is the demographic profile of patients undergoing PAO in our region. Most patients were non-athletic females presenting with symptomatic dysplasia at a relatively older age compared with many Western cohorts. Cultural and lifestyle factors, including lower participation in high-impact sports and potential delays in referral for hip preservation evaluation, may influence both the timing of diagnosis and clinical presentation in Middle Eastern populations. These demographic and lifestyle differences may also be associated with subtle variations in acetabular morphology or femoral coverage patterns, which could affect both the technical approach to PAO and postoperative outcomes. Awareness of these population-specific factors is important when interpreting results and comparing outcomes with Western or East Asian cohorts.

PAO is technically demanding but maintains a favourable safety profile. Systematic reviews and recent combined PAO–arthroscopy series report predominantly minor complications [8–10]. In our cohort, transient neuropraxia and minor intraoperative fractures did not compromise functional outcomes, reinforcing the safety of PAO when performed by experienced surgeons. Residual intra-articular pathology requiring subsequent arthroscopy occurred rarely, consistent with registry observations [16].

Collectively, our results reinforce three well-established principles: PAO provides reliable and sustained functional improvement [1, 2, 6]; durable radiographic correction correlates with long-term joint preservation [6, 11]; and careful patient selection based on cartilage status, age, and morphologic characteristics is essential [7]. These findings highlight that long-term success relies not only on surgical technique but also on individualized patient assessment and optimization.

Our observed 5.6% conversion to THA at a mean follow-up of 7.8 years is consistent with findings from large registry and cohort studies. A Danish national registry analysis of 1,501 hips undergoing PAO reported an overall THA conversion rate of 8.2%, with hip survivorship of approximately 81% at 15 years [14]. Similarly, a registry-based cohort of 1,385 hips demonstrated survivorship rates of 96% at five years and 90% at ten years following PAO [15]. Long-term survivorship studies further report approximately 86% native hip survival at ten years, with preoperative osteoarthritis severity and patient age identified as key predictors of failure [17, 18]. These data suggest that the low conversion rate observed in our cohort aligns with the durable joint preservation reported in large international series (Table 2).

In our cohort, both conversions to THA occurred in patients with preoperative Tönnis Grade 2 osteoarthritis, highlighting the impact of existing joint degeneration on PAO outcomes. Previous studies have consistently demonstrated that higher Tönnis grades, older age, and advanced chondral damage are significant predictors of failure after PAO [7, 17, 18]. While PAO provides durable joint preservation in patients with minimal or no osteoarthritis, those with moderate degenerative changes are at increased risk for early progression and THA conversion.

Careful patient selection is therefore essential. Indications for PAO in Tönnis Grade 2 hips typically include pain localized to the hip, limited radiographic osteoarthritis progression, preserved joint space in critical weight-bearing regions, and absence of severe femoral head deformity [7, 17]. Patients should be counseled regarding realistic functional outcomes and the potential need for future THA. Registry and long-term cohort data suggest that even in these higher-risk patients, PAO may provide several years of symptom relief and improved function, but the probability of eventual THA is substantially higher than in hips with Tönnis 0–1 [7, 18] (Tables 1 and 2).

**Table 1:** Comprehensive summary of patient, surgical, radiological, and functional outcomes with subgroup analyses and correlations (*n* = 36)

Domain	Parameter	Overall (*n* = 36)	Subgroup < 40y (*n* = 25)	Subgroup ≥ 40y (*n* = 11)	Effect Size/Correlation/*p*-value
Demographics	Age, mean ± SD (range)	34.6 ± 7.2 (19–49)	30.2 ± 5.1	42.5 ± 2.6	-
	Female, n (%)	28 (78%)	20 (80%)	8 (73%)	0.65
	BMI, mean ± SD (kg/m^2^)	24.3 ± 3.1	23.9 ± 2.8	25.1 ± 3.5	0.12
	Preoperative cartilage (Tönnis 0/1/2), n (%)	0: 13 (36%) 1: 17 (47%) 2: 6 (17%)	0: 13 (52%) 1: 9 (36%) 2: 3 (12%)	0: 2 (18%) 1: 6 (55%) 2: 3(27%)	0.15
Surgical Details	Operative time (min), mean ± SD	142 ± 25	140 ± 24	146 ± 26	P = 0.48 Additional acetabular correction had longer operative times (155 ± 22 vs. 138 ± 20 min, p = 0.01, Cohen’s d = 0.78)
	Blood loss (mL), mean ± SD	420 ± 85	400 ± 90	450 ± 70	r = 0.48 with time, *p* = 0.002
	Overall complications, n (%)	6 (16.7%)	3 (13.6%) All are Minor (2 transient LFCN neuropraxia, and 1 superficial infection)	3 (21%) All are Major, 2 conversions to THA and 1 Removal for hardware prominence	r = 0.42 with Tönnis grade, *p* = 0.01
Radiological Outcomes	LCEA (°) Preop	16.2 ± 4.3	16.5 ± 4.1	15.7 ± 4.7	0.63
	LCEA Postop	32.5 ± 3.6	33.0 ± 3.4	31.2 ± 3.8	0.19
	LCEA Last FU	31.8 ± 3.9	32.3 ± 3.7	30.5 ± 4.2	0.24
	Δ LCEA	+ 15.6 (95% CI 14.1–17.1)	+ 15.8° (95% CI 14.0 to 17.6)	+ 15.1° (95% CI 13.0 to 17.2)	0.61
	Acetabular Index Preop	22.8 ± 5.1	22.4 ± 5.0	23.5 ± 5.5	0.58
	Acetabular Index Postop	6.5 ± 3.4	6.0 ± 3.2	7.5 ± 3.7	0.26
	Acetabular index at final FU	7.2 ± 3.6°	7.0 ± 3.4	7.6 ± 3.9	0.66
	Δ AI	− 15.6 (95% CI − 17.2 to − 14.0)	− 15.8° (95% CI − 17.8 to − 13.8)	− 15.1° (95% CI − 17.4 to − 12.8)	0.58
	Coverage Angle Preop	55.1 ± 6.2	55.5 ± 6.0	54.2 ± 6.5	0.58
	Coverage Angle Postop	72.3 ± 5.4	73.1 ± 4.9	70.5 ± 6.3	0.24
	Coverage angle at final FU	71.5 ± 5.6°	72.0 ± 5.3	70.6 ± 6.0	0.42
	Δ Coverage angle	+ 16.4 (95% CI 14.8–18.0)	+ 16.6° (95% CI 14.5 to 18.7)	+ 15.9° (95% CI 13.4 to 18.4)	0.57
	ICC Interobserver	0.92 (0.88–0.95)	–	–	–
Functional Outcomes	HHS, mean ± SD	63.5 ± 11.2 → 89.6 ± 7.8	65.0 → 90.3	60.2 → 87.2	P = 0.24 The magnitude of LCEA correction correlated positively with HHS improvement r = 0.51, *p* < 0.001
	ΔHHS	+ 26.1 (95% CI 22.4–29.8)	+ 26.5 (95% CI 23.8 to 29.2)	+ 25.3 (95% CI 21.9 to 28.7)	P = 0.54
	WOMAC	42.1 → 14.3	43.0 → 13.8	40.5 → 15.5	P = 0.47 AI correction correlated with WOMAC improvement (r = 0.46, *p* = 0.002)
	Δ WOMAC	− 27.8 (95% CI − 31.2 to − 24.4)	− 29.2 (95% CI − 32.4 to − 26.0)	− 25.6 (95% CI − 30.1 to − 21.1)	P = 0.33
	HOS	61.4 → 86.7	63.2 → 87.5	57.5 → 85.0	P = 0.42
	ΔHOS	+ 25.3 (95%CI 21.9–28.7)	+ 25.8 (95% CI 22.9 to 28.7)	+ 24.2 (95% CI 20.5 to 27.9)	P = 0.49
	SF-36 PCS	38.2 → 52.7	39.0 → 53.5	36.5 → 50.8	P = 0.21
	Δ SF-36 PCS	+ 14.5 (95% CI 12.1–16.9)	+ 14.8 (95% CI 12.6 to 17.0)	+ 13.9 (95% CI 11.1 to 16.7)	P = 0.62
Additional Subgroup/Predictive Analysis	ΔHHS Tönnis 0–1 vs 2	27.0 ± 6.5 vs 18.5 ± 5.8	–	–	Higher Tönnis → lower ΔHHS, *p* < 0.01
	THA Conversion, n (%)	2 (5.6%)	0	2	Both Tönnis 2, LCEA correction < 12°
	Rehabilitation adherence	Full: 28, Partial: 6, Non: 2	–	–	Full adherence → better ΔHHS (r = 0.39, *p* = 0.02)

**Table 2:** Comparative summary of key PAO studies

Study	Year	N (hips/patients)	Mean/Range Follow-up (yrs)	Age/Gender	Complications (%)	Radiological Outcome	Functional Outcome	Operative Details/Notes	Key Findings
Swarup et al. [1]	2020	48	2–5	Adolescents, mixed gender	NR	LCEA improved; AI corrected	HHS improved (Δ ~ 25)	Standard PAO, single surgeon	Substantial functional improvement in borderline dysplasia
Kumar et al. [2]	2024	38	2 +	< 50y, mixed gender	10% minor	LCEA + 16°, AI − 14°	HHS + 26, WOMAC − 28	Minimally invasive PAO, single-surgeon	Significant radiological and functional improvement; short-term outcomes
Kołodziejczyk et al. [3]	2022	56	3–7	Adolescents/young adults	12%	LCEA corrected; AI improved	HHS + 24	Childhood osteotomy improved PAO results	Early osteotomy improved PAO outcomes
Chang et al. [4]	2023	22	5–8	Multiple epiphyseal dysplasia	9%	LCEA + 14°	HHS + 22	Standard Bernese PAO	Favorable outcomes despite complex hip morphology
Tan et al. [6]	2022	72	10	Adult	8% major/minor	LCEA + 15°; AI − 15°	HHS + 25, WOMAC − 26	Standard PAO	Durable functional improvement and survivorship at 10 yrs
Novais et al. [7]	2023	44	7–12	≥ 40y	11%	LCEA + 13°; AI − 12°	HHS + 22–25	Older patients, preserved cartilage	Older patients achieve meaningful improvement if cartilage preserved
Jakobsen et al. [14]	2023	1,501	15–24	Mixed adult	18% (secondary surgery)	Radiographic preservation 81% at 15y	PROM improved	Registry study	Large-scale evidence of long-term joint preservation
Smith et al. [15]	2024	Meta-analysis	5–20	Mixed	4–32%	LCEA + 14–16°	HHS + 20–28	Multiple cohorts	Benchmark survivorship rates; supports efficacy of PAO
Current study	2026	36	7.8 ± 1.2	34.6 ± 7.2, 78% F	16.7% (minor 8%, major 8.3%)	LCEA + 15.6° (95% CI 14.1–17.1); AI − 15.6° (− 17.2 to − 14.0)	HHS + 26.1 (95% CI 22.4–29.8); WOMAC − 27.8 (− 31.2 to − 24.4)	Bernese PAO; operative time 142 ± 25 min; blood loss 420 ± 85 mL	Comparable functional/radiological improvements; low THA rate; regional specificity noted (Middle Eastern cohort, lower BMI)

Strengths of this study include its prospective design, minimum seven year follow-up, and comprehensive evaluation of both PROMs and radiographic outcomes. Limitations include the single-center nature, modest sample size, and limited THA events, which restrict causal inference regarding osteoarthritis progression. The relatively modest sample size and the limited number of THA conversion events represent an important limitation of the present study. These factors may reduce the statistical stability of multivariate regression models and introduce the potential for overfitting. Therefore, predictors of survivorship identified in this cohort should be interpreted with caution and validated in larger multicenter studies. Nevertheless, the sustained functional and radiographic durability observed mirrors outcomes in larger international cohorts [6, 7].

**Fig. 3:**
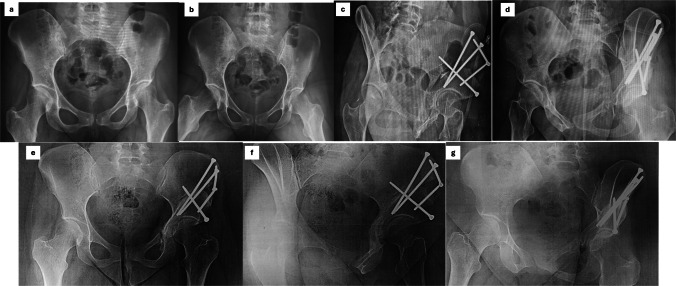
A 41-year-old female with symptomatic adult acetabular dysplasia, predominantly affecting the left hip, treated with PAO. **a**, **b** Preoperative radiographs demonstrating acetabular dysplasia with reduced LCEA and increased AI. **c**, **d** Immediate postoperative radiographs showing adequate acetabular reorientation with correction of both LCEA and AI, stabilized using five 4.5-mm cortical screws. **e–g** Final follow-up radiographs demonstrating maintained acetabular correction, complete union

In summary, PAO in this Middle Eastern population achieved durable radiographic correction, substantial functional improvement, and low mid-term THA conversion. When applied to carefully selected patients considering cartilage status, age, BMI, and regional morphology, PAO effectively restores hip biomechanics and may contribute to delayed arthroplasty [2, 6, 7] (Figs. 1, 2 and 3).

## Conclusion

In this single-center Middle Eastern cohort, periacetabular osteotomy provided sustained improvements in pain, function, and quality of life over a minimum seven-year follow-up. Radiographic correction was durable, with excellent interobserver reliability, and the overall complication rate was low. Conversion to total hip arthroplasty occurred in a small proportion of patients, primarily those with advanced preoperative cartilage degeneration. These results confirm that, when performed in appropriately selected patients, PAO is a reliable joint-preserving intervention that restores hip biomechanics, delays osteoarthritis progression, and maintains long-term functional outcomes. Early referrals, meticulous surgical techniques, and structured rehabilitation are essential to achieve optimal patient outcomes.

## Data Availability

No datasets were generated or analysed during the current study.
